# Characterization of Tumor Microenvironment in Lung Adenocarcinoma Identifies Immune Signatures to Predict Clinical Outcomes and Therapeutic Responses

**DOI:** 10.3389/fonc.2021.581030

**Published:** 2021-03-05

**Authors:** Donglai Chen, Yifei Wang, Xi Zhang, Qifeng Ding, Xiaofan Wang, Yuhang Xue, Wei Wang, Yiming Mao, Chang Chen, Yongbing Chen

**Affiliations:** ^1^ Department of Thoracic Surgery, Shanghai Pulmonary Hospital, Tongji University, School of Medicine, Shanghai, China; ^2^ Department of Thoracic Surgery, The Second Affiliated Hospital of Soochow University, Suzhou, China; ^3^ Stem Cell Translational Research Center, Tongji Hospital, Tongji University School of Medicine, Shanghai, China; ^4^ Department of Neurology, Tongji Hospital, Tongji University School of Medicine, Shanghai, China; ^5^ Department of Thoracic Surgery, Suzhou Kowloon Hospital, Shanghai Jiaotong University School of Medicine, Suzhou, China

**Keywords:** lung adenocarcinoma, tumor microenvironment, signature, survival, therapeutic response

## Abstract

**Background and Objective:**

Increasing evidence has elucidated the clinicopathological significance of individual TME component in predicting outcomes and immunotherapeutic efficacy in lung adenocarcinoma (LUAD). Therefore, we aimed to investigate whether comprehensive TME-based signatures could predict patient survival and therapeutic responses in LUAD, and to assess the associations among TME signatures, single nucleotide variations and clinicopathological characteristics.

**Methods:**

In this study, we comprehensively estimated the TME infiltration patterns of 493 LUAD patients and systematically correlated the TME phenotypes with genomic characteristics and clinicopathological features of LUADs using two proposed computational algorithms. A TMEscore was then developed based on the TME signature genes, and its prognostic value was validated in different datasets. Bioinformatics analysis was used to evaluate the efficacy of the TMEscore in predicting responses to immunotherapy and chemotherapy.

**Results:**

Three TME subtypes were identified with no prognostic significance exhibited. Among them, naïve B cells accounted for the majority in TMEcluster1, while M2 TAMs and M0 TAMs took the largest proportion in TMEcluster2 and TMEcluster3, respectively. A total of 3395 DEGs among the three TME clusters were determined, among which 217 TME signature genes were identified. Interestingly, these signature genes were mainly involved in T cell activation, lymphocyte proliferation and mononuclear cell proliferation. With somatic variations and tumor mutation burden (TMB) of the LUAD samples characterized, a genomic landscape of the LUADs was thereby established to visualize the relationships among the TMEscore, mutation spectra and clinicopathological profiles. In addition, the TMEscore was identified as not only a prognosticator for long-term survival in different datasets, but also a predictive biomarker for the responses to immune checkpoint blockade (ICB) and chemotherapeutic agents. Furthermore, the TMEscore exhibited greater accuracy than other conventional biomarkers including TMB and microsatellite instability in predicting immunotherapeutic response (*p* < 0.001).

**Conclusion:**

In conclusion, our present study depicted a comprehensive landscape of the TME signatures in LUADs. Meanwhile, the TMEscore was proved to be a promising predictor of patient survival and therapeutic responses in LUADs, which might be helpful to the future administration of personalized adjuvant therapy.

## Introduction

Lung adenocarcinoma (LUAD) is the commonest histological type of all lung cancers, accounting for approximately 50% of them (1, 2). Nowadays, surgical resection remains as the standard treatment for early-stage LUADs. Meanwhile, chemotherapy plays an important role in LUAD patients at all stages of the disease. Recently, blockade of immune checkpoint has been proved as a promising therapy for patients with LUADs (3, 4) which seems to be an alternative to conventional chemotherapy. Nevertheless, the fact that most patients do not derive any benefit from PD-1/PD-L1 blockade combined with the risk of serious immune-related adverse events (5, 6) and the significant up-front costs, underscore the need for developing accurate tools for predicting therapeutic response to chemotherapy and immune checkpoint blockade (ICB) (7).

Nowadays, an increasing body of literature suggests a crucial role for the tumor microenvironment (TME) in cancer progression and therapeutic responses (8–11). The TME context in LUADs has been reported not only to reflect the potential benefits from treatment (12–14), but also to predict patient survival (15, 16), which includes tumor-infiltrating lymphocytes (e.g., CD8+ T cells, CD4+ T cells), tumor-associated macrophages (TAMs), cancer-associated fibroblasts (CAFs), and other cell types. With the introduction of computational methods to assess the abundance of cells infiltrating in the TME, several studies using these methodologies have explored the clinical utility of TME context (13, 17–19). However, the comprehensive landscape of TME infiltrates and its predictive power for therapeutic responses in LUADs have not been fully investigated.

In our present study, two previously proposed computational algorithms (20, 21) were employed to estimate the fractions of 23 immune and stromal cells based on LUAD gene expression profiles from The Cancer Genome Atlas (TCGA) database. The TME infiltrating patterns of LUAD samples were investigated and correlated with both transcriptomic characteristics and clinicopathological features. Unsupervised clustering was applied to quantify the TME infiltrating patterns which were calculated as a TMEscore. Consequently, the TMEscore was proved to be a promising prognostic biomarker and a robust predictive factor for the therapeutic responses in LUADs.

## Materials and Methods

### LUAD Datasets and Preprocessing

The transcriptomic dataset of LUAD from TCGA database was downloaded from the UCSC Xena browser (https://xenabrowser.net/datapages/). LUAD patients without survival information were removed from further evaluation, among whom 499 were available to construct the TMEscore. Data of somatic mutations (MuSE Variant Aggregation and Masking) were downloaded from TCGA database, which included 567 LUAD specimens. Somatic mutation data, transcriptomic data and survival information were available in 493 of the 499 specimens, of which clinical characteristics were accessible in 474. The raw data in TCGA dataset generated from Illumina were processed using the lumi software package according to a previous study (8).

The microarray data (GSE68465) generated by Affymetrix were obtained from the Gene Expression Omnibus (https://www.ncbi.nlm.nih.gov/geo/) (GEO) as a validation dataset, which included 422 LUAD specimens with expression profiles and clinical outcomes available. The raw data for the dataset from Affymetrix were processed using the Range Migration Algorithm (RMA) for background adjustment in the Affy software package (22). The RMA was used to perform background adjustment, quantile normalization, and final summarization of oligonucleotides per transcript using the median polish algorithm. The detailed information of the LUAD datasets is listed in **Supplementary Table 1**.

### Assessment of Infiltrating Cells in TME

To calculate the proportions of immune cells in the LUAD samples, we used the CIBERSORT (cell type identification by estimating relative subset of known RNA transcripts) algorithm (20) and the LM22 gene signature, which allows for highly sensitive and specific discrimination of 22 human immune cell phenotypes (8). CIBERSORT is a deconvolution algorithm that uses a set of reference gene expression values (a signature with 547 genes) considered a minimal representation for each cell type and, based on those values, infers different cell type proportions in tumor samples using support vector regression (8). Gene expression profiles were prepared using standard annotation files, and data were uploaded to the CIBERSORT web portal (http://cibersort.stanford.edu/), with the algorithm run using the LM22 signature and 1,000 permutations. Proportions of stromal cells were estimated by applying the Microenvironment Cell Populations (MCP)-counter method, which allows for robust quantification of the absolute abundance of immune and stromal cell populations in heterogeneous tissues from transcriptomic data (21).

### Consensus Clustering for TME-Infiltrating Cells

Hierarchical agglomerative clustering (based on Euclidean distance and Ward’s linkage) was employed to group the samples with qualitatively different TME cell infiltration patterns. Unsupervised clustering methods (K-means) (23) for dataset analysis were used to identify TME patterns and classify patients for further analysis. A consensus clustering algorithm was applied to determine the number of clusters (8), which was repeated 1000 times to ensure the stability of classification using the ConsensuClusterPlus R package (24).

### Generation and Analysis of TME Gene Signatures

To identify genes associated with TME cell infiltrating patterns, we classified patients into three TMEclusters. Differentially expressed genes (DEGs) among these three groups were determined using the R package limma (25). DEGs among different TME patterns were determined by significance criteria (adjusted *p* value < 0.05; |logFC|>0.58). An unsupervised clustering method (K-means) for analysis of DEGs was employed to classify patients into two groups for further analysis. TME signature genes were then obtained using the random forest classification algorithm to screen redundant genes. Gene-annotation enrichment analysis using the clusterProfiler R package (26) was performed on TME signature genes. Gene Ontology (GO) terms were identified with a strict cutoff of *p* < 0.01 and false discovery rate (FDR) of less than 0.05.

### Establishment of TME Scores

Cox regression model was applied to assess the prognostic value of each signature gene which was classified according to its Cox coefficient. A method similar to gene expression grade index (24) was used to define the TMEscore of each patient:

$$TMEscore=\Sigma \;log_{2}(X+1)-\Sigma \;log_{2}(Y+1)$$

where X is the expression level of genes whose Cox coefficient is positive, and Y is the expression level of genes whose Cox coefficient is negative. The cut-off values of each dataset were evaluated based on the association between patient overall survival (OS) and TMEscore in each separate dataset using the survminer package (8). The R package MaxStat (27) which iteratively tests all possible cut points to find the one achieving the maximum rank statistic, was used to dichotomize TMEscore, and patients were then divided into low- and high-TMEscore subgroups.

### Analysis of Tumor Mutation Profiles

Somatic mutation data were obtained from the publicly available TCGA database. Notably, one sample with merely silent mutation was excluded from the aforementioned 493 samples in our analysis. We prepared the Mutation Annotation Format (MAF) of somatic variants, and implemented the R package Maftools (https://bioconductor.org/packages/release/bioc/html/maftools.html) which provides a multiple of analysis modules to perform the visualization process (28) to display somatic landscape. In addition, the R package SomaticSignatures (https://bioconductor.org/packages/release/bioc/html/SomaticSignatures.html) was used to characterize the mutation signatures of the LUAD samples (29). Mutational signatures were extracted using 96 nonnegative components (single-base somatic substitutions and their immediate sequence context) and compared to the validated consensus mutational signatures in the Catalogue Of Somatic Mutations In Cancer (COSMIC) (30), version 2 (https://cancer.sanger.ac.uk/cosmic/signatures_v2) to identify the set of COSMIC mutational signatures in TCGA datasets (31). Moreover, the estimation of TMB in LUAD samples was conducted according to a previous study (32).

### Predictive Value of TMEscore to Estimate Therapeutic Effect

Tumor Immune Dysfunction and Exclusion (TIDE) (http://tide.dfci.harvard.edu/) (33), a computational method to predict ICB response based on melanoma patients who underwent anti-PD-1 or anti-CTLA-4 agent, was used to investigate the predictive value of TMEscore for immunotherapy. TIDE uses a set of gene expression markers to estimate two distinct mechanisms of tumor immune evasion, including dysfunction of tumor infiltrating cytotoxic T lymphocytes (CTL) and exclusion of CTL by immunosuppressive factors (34). Patients with higher TIDE score have a higher chance of antitumor immune escape, thus exhibiting lower response rate of ICB treatment (33). The TIDE score was shown to have a higher accuracy than PD-L1 expression level and tumor mutation burden (TMB) in predicting survival outcome of cancer patients treated with ICB agents (34–37). The R package MaxStat (27) was also employed to dichotomize the TMB level. The R package pRRophetic (38) was used to determine whether TMEscore could accurately predict clinical chemotherapeutic responses.

### Immunohistochemistry Staining

LUAD samples resected from a cohort of chemo- and/or radio-naïve patients (**Supplementary Table 2**) were obtained from the Second Affiliated Hospital of Soochow University, which was approved by the Institutional Review Board (IRB NO.JD-HG-2020-09). The chairperson of the ethics committee waived the need for patient consent. The sections of tumor tissues were firstly deparaffinized and rehydrated. Endogenous peroxidase was then quenched using 10% H2O2 for 10 min at room temperature. Subsequently, nonspecific proteins were blocked with 10% goat serum for 1 h. Afterwards, the sections were rinsed and incubated with anti-BTK (YM0083, Immunoway; diluted 1: 400) overnight at 4°C. The DAB Horseradish Peroxidase Color Development Kit (Beyotime, China) was used for color development. Finally, the sections were counterstained with hematoxylin and mounted.

As described in our previous study (39), the staining index was calculated as a product of staining intensity (negative = 0, weak = 1, moderate = 2, and strong = 3) multiplied by staining extent (0% = 0, 1%–10% = 1, 11%–50% = 2, and > 50% = 3). A final score of 0–2 indicated low BTK expression, and a score of > 2 indicated high BTK expression.

### Statistical Analysis

For comparisons of two subgroups, unpaired Student t tests was used to estimate statistical significance for normally distributed variables, and Wilcoxon rank-sum test was used for analyzing non-normally distributed variables. To identify significant genes in the DEG analysis, Benjamini-Hochberg method was applied to converting the *p* values to FDRs (40). The Kaplan-Meier method was used to generate survival curves for the subgroups in each dataset, and the Log-rank test was used to determine the statistical significance of differences. The hazard ratios for univariate analyses were calculated using a univariate Cox proportional hazards regression model. A multivariate Cox regression model was used to determine independent prognostic factors. The R package pROC (41) was used to plot and visualize receiver operating characteristic (ROC) curves to calculate the area under the curve (AUC) and confidence intervals to evaluate the diagnostic accuracy of TMB and TMEscore. For comparison of AUCs, likelihood ratio test for two correlated ROC curves was used. All statistical analyses were conducted using R (https://www.r-project.org/) or SPSS software (version 25.0). A two-tailed *p*-value < 0.05 was considered statistically significant.

## Results

### Characterization of TME in LUADs and Distinct Patterns of TME Subtypes

**Figure 1:**
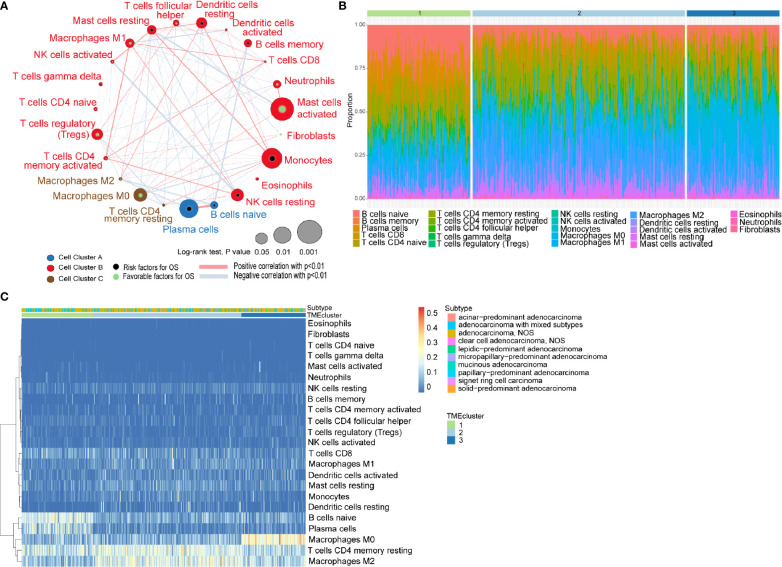
Characterization of TME in LUADs and distinct patterns of TME subtypes. **(A)** Cellular interaction of tumor microenvironment (TME) cell types in LUADs; **(B)** Barplot showing the specific 23 immune fractions represented by various colors in each TMEcluster; **(C)** Unsupervised clustering of TME cell types and histologic subtypes for LUAD patients.

The general flowchart of our study is shown in **Supplementary Figure 1A**. A TCGA dataset comprised of 499 patients with their transcriptomic data were included in our initial analysis. By applying CIBERSORT algorithm and MCP-counter method, we obtained the proportions of 23 different immune and stromal cells in the 499 samples (**Supplementary Figure 1B**). Meanwhile, a TME cell network was used to depict the comprehensive landscape of tumor-immune cell interactions and their effects on the OS of patients with LUADs (**Figure 1A** and **Supplementary Table 3**).

Unsupervised learning using K-means algorithm was used on the dataset to perform group clustering, which identified *K*=3 according to the elbow method and gap statistic (**Supplementary Figures 2A, B**). To identify the aforementioned optimal cluster number, we assessed clustering stability using the ConsensusClusterPlus R package which displayed the clustering stability using 1,000 iterations of hierarchical clustering. The consensus matrix supported the existence of three robust clusters of LUADs (**Supplementary Figure 2C**). As shown in **Figures 1B, C**, the proportions of infiltrating immune cells and histologic subtypes differ significantly among the three TME subtypes. We found that naïve B cells accounted for the majority in TMEcluster1, while M2 TAMs and M0 TAMs took the largest proportion in TMEcluster2 and TMEcluster3, respectively. However, log-rank test revealed no significant difference in survival among different TMEclusters (*p* = 0.45) (**Supplementary Figure 2D**).

### Construction and Validation of the TMEscore in Different LUAD Datasets

A total of 3395 DEGs among the three TME clusters were determined by significance criteria (adjusted *p* value < 0.05; |logFC| > 0.58) as implemented in the R package limma (**Supplementary Figure S3A**). An unsupervised clustering method (K-means) for analysis of DEGs was then employed to classify the patients into two groups (**Supplementary Figure 3B**). Among these DEGs, 217 TME signature genes were obtained using the random forest classification algorithm, on which Gene-annotation enrichment analysis using the clusterProfiler R package (26) was performed. Consequently, the data indicated that these TME signature genes significantly enriched in pathways associated with T cell activation, lymphocyte proliferation and mononuclear cell proliferation (**Supplementary Figure 3C**).

**Figure 2:**
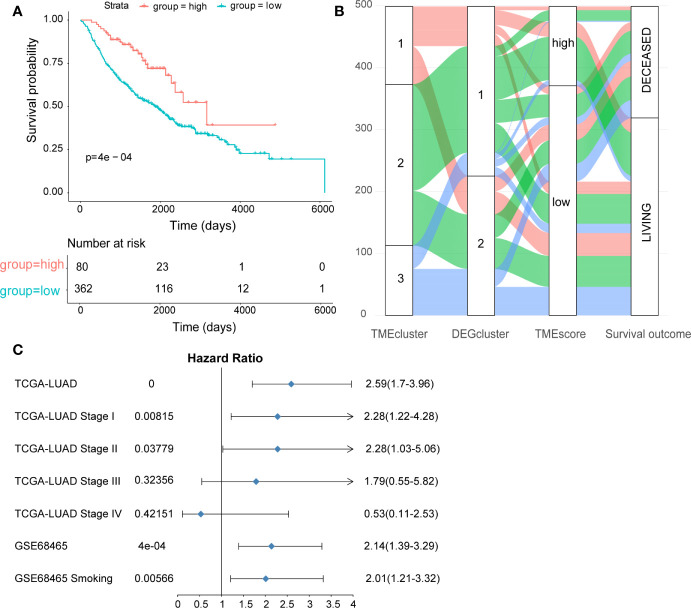
Construction and validation of the TMEscore in different LUAD datasets. **(A)** Kaplan-Meier curves of high- and low-TMEscore subgroups in the entire TCGA cohort; **(B)** Alluvial diagram showing the relationships among TME subtypes and TMEscore subgroups as well as clinical outcomes; **(C)** Forest plot showing the prognostic value of TMEscore in different datasets.

Cox regression model was used to assess the prognostic value of each signature genes according to the Cox coefficient, by which the TMEscore was established for each patient. As shown in **Figure 2A**, the TMEscore could effectively distinguish significantly different OS in the entire cohort. Notably, patients with high TMEscore (n = 120) had significantly better survival than those with low TMEscore (n = 354) (*p* < 0.0001). Additionally, we visualized the relationships of TME subtypes and TMEscore subgroups as well as patient outcomes using an alluvial diagram (**Figure 2B**). Moreover, TMEscore remained efficient in stratifying the patients with early-stage disease (stage I-II) into different OS (**Supplementary Figures 4A, B**), which exhibited its value in the external validation cohort from the GEO database as well (**Supplementary Figure 4C**). To be noted, even for smokers in the validation cohort, significantly different OS was also observed among the subgroups stratified by the TMEscore (**Supplementary Figure 4D**). A forest plot was used to summarize the predictive value of TMEscore in different patient cohorts (**Figure 2C**).

### Association Between TMEscore and Cancer Somatic Genomes

**Figure 3:**
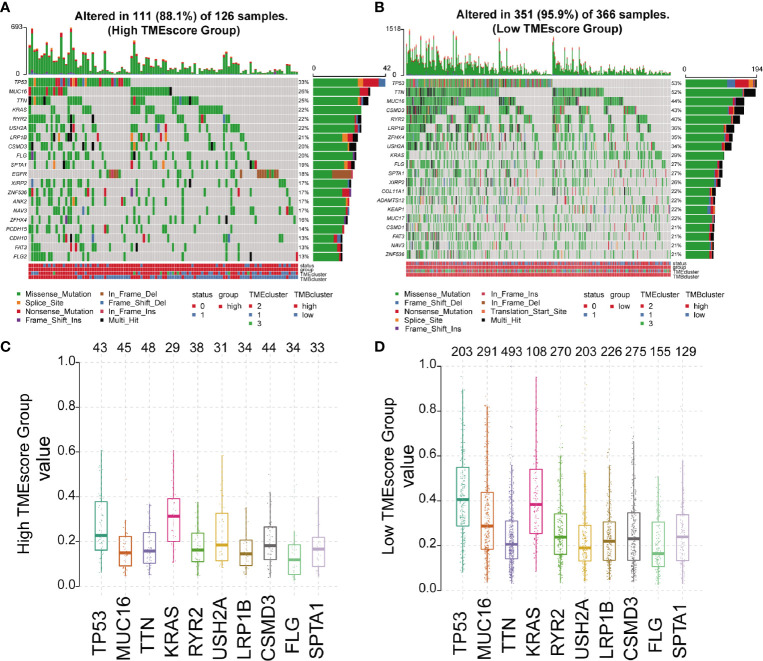
Mutation profiles of different TMEscore subgroups. **(A, B)** Waterfall plots exhibiting the mutation profiles of patients with high/low-TMEscore in which various colors with annotations at the bottom represented the different mutation types and tumor mutation burden; **(C, D)** Boxplots showing the mutation frequency of the 10 most frequently mutated genes in high- and low-TMEscore subgroups.

We analyzed the somatic variations of the 492 LUAD samples in TCGA database which revealed missense mutation as the leading type of single nucleotide variant (SNV) (**Supplementary Figure 5A**). Meanwhile, SNV was identified as the major variant in LUADs that occurred more frequently than insertion or deletion (**Supplementary Figure 5B**). In addition, it was observed that C>A was the predominant SNV type in LUADs (**Supplementary Figure 5C**). Besides, we calculated the TMB and showed the mutation type with different colors in LUAD samples (**Supplementary Figures 5D, E**), as well as the top 10 mutated genes in LUADs with ranked percentages (**Supplementary Figure 5F**). We also mapped the landscape of mutation profiles among the three TMEclusters, which characterized the mutation types of the frequently mutated genes (**Supplementary Figure 6**). Waterfall plots were then used to exhibit the mutation profiles of patients with high/low-TMEscore in which various colors with annotations at the bottom represented the different mutation types and TMB levels (**Figures 3A, B**). Meanwhile, boxplots were applied to showing the mutation frequency of each frequently mutated genes in high- and low-TMEscore subgroups, respectively (**Figures 3C, D**).

**Figure 4:**
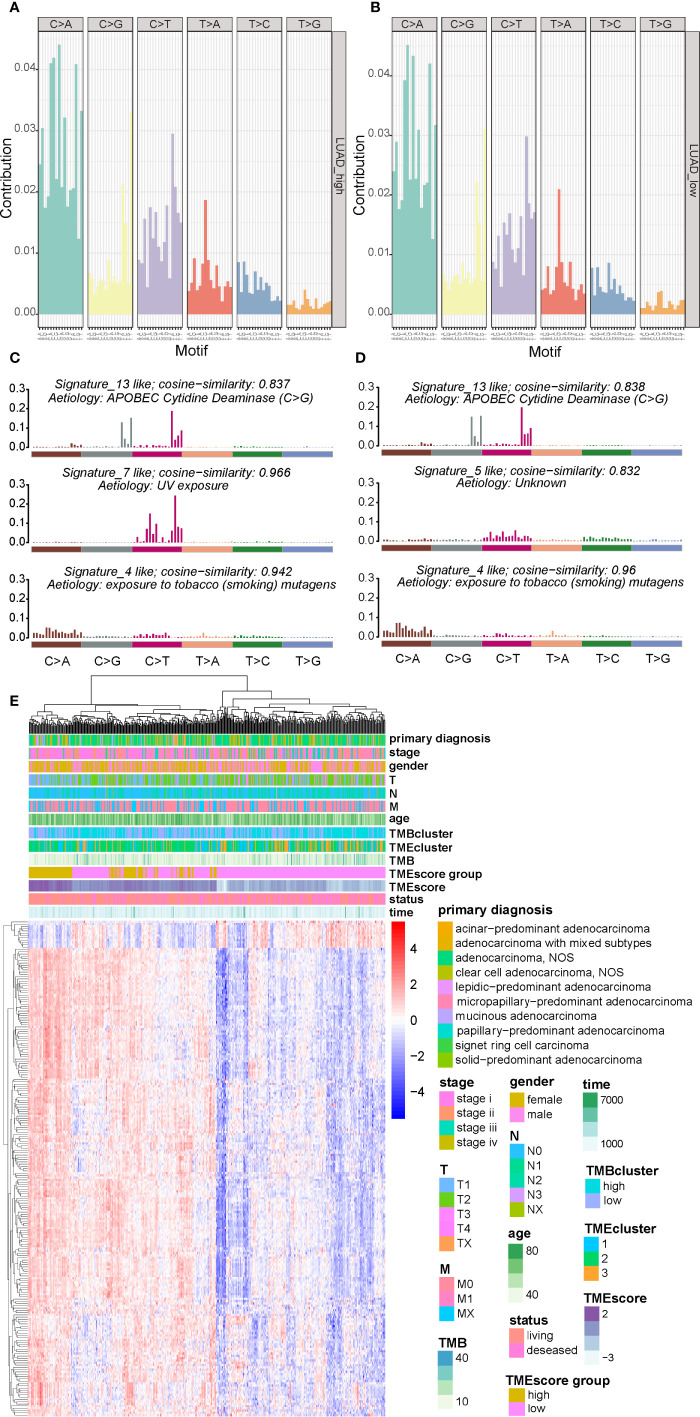
Visualization of mutational signatures and TME signatures in TME subgroups. **(A, B)** Mutation spectra showing the 96 substitution classification defined by the substitution class and sequence context immediately 3’ and 5’ to the mutated base in the high- and low-TME subgroups. **(C, D)** Barplot showing the differential mutation signatures between the high- and low-TME subgroups. The y-axis indicates exposure of 96 trinucleotide motifs to overall signature. The plot title indicates best match against validated COSMIC signatures and cosine similarity value along with the proposed etiology. **(E)** Unsupervised analysis and hierarchical clustering of the 217 TME signature genes and their associations with clinicopathological characteristics.

There are six classes of base substitution—C>A, C>G,C>T, T>A, T>C, T>G(all substitutions are referred to by the pyrimidine of the mutated Watson–Crick base pair)—and as we incorporated information on the bases immediately 5’ and 3’ to each mutated base, there are 96 possible mutations in this classification (30). This 96 substitution classification is particularly useful for distinguishing mutational signatures that cause the same substitutions but in different sequence contexts (30). A previous study (30) applied this approach to the 30 cancer types and revealed 21 distinct validated mutational signatures. Each mutational signature was characterized by different substitutions and was associated with epidemiological and biological features of particular cancer types. Therefore the frequency distribution of the 96 mutations based on the six classes of base substitution were analyzed in both TMEscore subgroups (**Figures 4A, B**). Moreover, by comparing the extracted signatures from our samples with those in COSMIC, we identified different mutational signatures between the two TMEscore subgroups. The identified signatures in both groups similarly showed a strong resemblance to COSMIC signature 13 and COSMIC signature 4 (**Figures 4C, D**). A high similarity to COSMIC signature 7 was also found in the high-TMEscore subgroup (**Figure 4C**). Meanwhile, a mutational signature similar to COSMIC signature 5 was present in the low-TMEscore subgroup (**Figure 4D**). As reported previously (30), COSMIC signature 13 and COSMIC signature 4 were associated with APOBEC and smoking, respectively, while COSMIC signature 7 was mainly associated with ultraviolet light.

A genomic landscape of the LUADs was thereby plotted by integrating the TMB information and clinical characteristics with TME profiles including the 217 TME signature genes and TMEscore (**Figure 4E**).

### Predictive Value of TMEscore as a Biomarker for Therapeutic Effect

**Figure 5:**
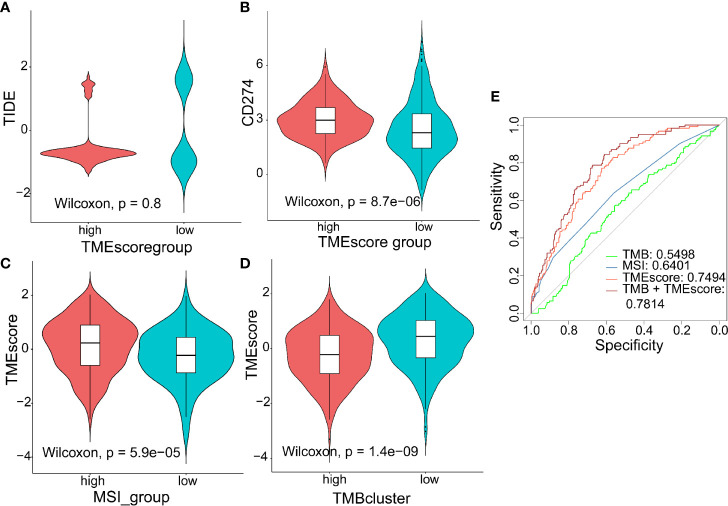
Predictive value of TMEscore as a biomarker for immunotherapy. **(A, B)** Violin plot showing TIDE score and PD-L1 level between the high- and low-TMEscore subgroups; **(C, D)** Violin plot showing TMEscores in groups with different microsatellite instability (MSI) status and with different TMB levels; **(E)** ROC curves to compare the accuracy of TMB, MSI and TMEscore in predicting responses to immunotherapy.

**Figure 6:**
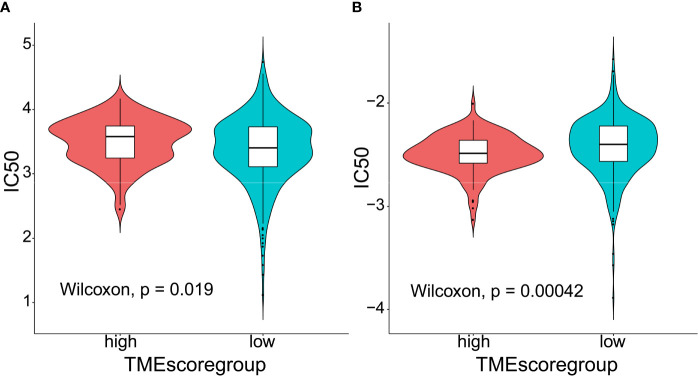
Predictive value of TMEscore as a biomarker for chemotherapy. **(A)** Violin plot comparing IC50 of cisplatin between the high- and low-TMEscore subgroups; **(B)** Violin plot comparing IC50 of gemcitabine between the high- and low-TMEscore subgroups.

TIDE to evaluate TMEscore as a predictor of immunotherapy, interestingly, no significant difference (*p* = 0.8) was observed in TIDE score between the high- (n = 120) and low-TMEscore (n = 354) subgroups (**Figure 5A**). However, we observed significantly different PD-L1 expression levels between the high- and low-TMEscore subgroups (*p* < 0.001) (**Figure 5B**). Since microsatellite instability (MSI), the spontaneous loss or gain of nucleotides from repetitive DNA tracts, is a promising predictive biomarker for patient survival and response to immunotherapy (42, 43), TMEscore was compared between the two subgroups stratified by MSIscore which was calculated by TIDE (44). As shown in **Figure 5C**, TMEscore in the high-MSI subgroup (n=314) was significantly higher than that in the low-MSI one (n = 160) (*p* < 0.001). Meanwhile, TMEscore in the high-TMB subgroup (n = 257) was significantly lower than that in the low-TMB one (n = 217) (*p* < 0.001) (**Figure 5D**). Furthermore, the AUC indicated that TMEscore was superior to TMB alone in predicting response to ICB (*p* < 0.001) which exhibited greater accuracy in combination with TMB (**Figure 5E**). Additionally, comparison of the 50% inhibitory concentration (IC50) of chemotherapy drugs indicated that the low-TMEscore subgroup had higher sensitivity to cisplatin (*p* = 0.019) (**Figure 6A**) while the high-TMEscore subgroup was prone to the benefits from gemcitabine (*p* = 0.00042) (**Figure 6B**).

## Discussion

Our findings indicated that assessment of the immune and stromal statuses *via* the TME signature provided a potent predictor of survival in early-stage patients with LUAD and a promising biomarker for therapeutic responses as well. Based on the DEGs and GO enrichment analysis, we observed that the TME signature genes significantly enriched in pathways mainly associated with lymphocyte activation and proliferation. Moreover, missense mutation was identified as the leading type of SNV which was identified as the major variant in LUADs. Meanwhile, the established TMEscore could stratify the patient cohort from TCGA database into two subgroups with distinct mutation profiles and COSMIC signatures. A genomic landscape of the LUADs was also characterized by integrating the TMB information and clinical characteristics with TME profiles. To our best knowledge, the present study is the first bioinformatics-based study to comprehensively investigate the associations among clinicopathological features, the mutation spectra and the TME profiles of LUADs, which also developed a computational algorithm and demonstrated its predictive value for both ICB and chemotherapeutic agents.

Previously, Yue et al. (45) identified a prognostic gene signature associated with TME, and validated its predictive accuracy for OS in LUAD patients. However, they omitted to analyze whether the signature could predict therapeutic responses to ICB or chemotherapy. In their study, univariate Cox regression analysis was initially employed to screen out 23 prognostic TME-related DEGs. Afterwards, Yue et al. (45) used selection operator (LASSO) and multivariate Cox regression analyses to identify three key genes for constructing a prognostic model, in which the analytical methods were different from ours. Interestingly, there are five common DEGs between their 23 TME-related DEGs and our 217 signature genes, including BTK, CCDC69, CD33, CD52, and LY86. Among the five common DEGs, only BTK was selected out as a signature gene in both their and our studies. We therefore validated the prognostic impacts of the five DEGs using the LUAD cohort from TCGA database (**Supplementary Figures 7A–E**). Additionally, the role of BTK as a prognostic factor was also validated in our domestic cohort using IHC (**Supplementary Figure 7F**). Similar to our data, Bi et al. (46) who performed a study based on TCGA mining also found that BTK was an immune-related gene and a promising prognostic factor for LUAD. Recently, Tan et al. (47) performed a bioinformatics study to characterize the immune landscape of LUADs, in which they divided the patients with LUAD into two immunophenotypes based on the tumor microenvironment. The two immunophenotypes were denoted as the Active Immune Class and Exhausted Immune Class. The former showed significant IFN, T‐cells, M1 macrophage signatures, and better prognosis, while the latter presented an exhausted immune response with activated stromal enrichment, M2 macrophage signatures, and immunosuppressive factors such as WNT/transforming growth factor‐β. In addition, Tan and his colleagues (47) identified their developed Immune Class as a useful tool to predict the response to ICB. However, merely 32 patients in a metastatic melanoma cohort were included in their prediction of PD-L1 inhibitor response. In addition, the relationships of clinicopathological characteristics and immunophenotypes were not comprehensively characterized, neither were the somatic landscape and COSMIC signatures. Additionally, Huang et al. (48) identified two LUAD subtypes with specific immune and metabolic state based on the bioinformatic analyses of TME, who constructed a TME score to predict TME phenotypes in LUADs. Interestingly, they constructed the TME score using the principal component analysis algorithm based on the TME signature genes, which could represent the signature of the two TME clusters in their study (48). Differently, their TME score was applied to evaluating the expression patterns of immune-associated genes in LUADs, which merely exhibited diagnostic value. It remained unknown whether the TME score raised by Huang et al (48) could accurately predict patient survival and therapeutic responses to ICB or chemotherapy. Meanwhile, the data of somatic variations in LUADs were unavailable in their study.

Hitherto, several bioinformatics studies (34, 47, 49, 50) have identified the immune-related signatures as a prognostic biomarker in LUADs. However, the roles of immunophenotype-derived signatures in predicting response to chemotherapy have not been fully clarified. Notably, a number of clinical investigations (51–53) have highlighted adjuvant chemotherapy as a prognostic factor for improved survival in patients with stage IB LUADs. Under the circumstances, our study not only proved TMEscore as a survival-related predictor, but also identified its potential in stratifying patients with distinct sensitivity to different chemotherapy regimens. According to our analysis, low-TMEscore subgroup had higher sensitivity to cisplatin while high-TMEscore group was more likely to respond to gemcitabine, which offered insights into the administration of personalized adjuvant therapy.

There are some limitations that should not be ignored in our study: 1) lack of domestic sequencing data to validate the associations between the TME infiltrating patterns and clinicopathological characteristics as well as mutation spectra; 2) lack of external validation cohort to confirm the roles of TMEscore in predicting therapeutic responses; 3) possessing predictive power though, the chosen set of signature genes are not necessarily valid in terms of their biological significance. For instance, the differences in IC50 scores between high- and low-TMEscore subgroups in **Figure 6** do not seem to be obvious although statistically significant differences were obtained. It raises the possibility that the observed differences may not be biologically important; however, they are statistically significant due to a large number of samples included in the analyses. More prospective clinical trials are warranted to verify the potentials of TMEscore in predicting patient outcomes and response to both chemotherapeutic agents and ICB.

In conclusion, our present study depicted a comprehensive landscape of the TME signatures in LUADs. Meanwhile, the TMEscore was proved to be a promising predictor of patient survival and therapeutic responses in LUADs, which might be helpful to the future administration of personalized adjuvant therapy.

## Data Availability Statement

The datasets presented in this study can be found in online repositories. The names of the repository/repositories and accession number(s) can be found in the article/**Supplementary Material**.

## Ethics Statement

Ethical review and approval were not required for the study on human participants in accordance with the local legislation and institutional requirements. Written informed consent for participation was not required for this study in accordance with the national legislation and the institutional requirements.

## Author Contributions

DC, YW, XZ, and QD: conceptualization, formal analysis, and writing original draft. XW, YX, and WW: data curation, methodology, and formal analysis. YM: software, methodology, supervision, and reviewing the draft. YC and CC: supervision, reviewing, and editing the draft. All authors contributed to the article and approved the submitted version.

## Funding

Jiangsu Key Research and Development Plan (Social Development) Project (BE2020653); Suzhou Key Discipline for Medicine (SZXK201803); Suzhou Key Laboratory of Thoracic Oncology (SZS201907); Municipal Program of People's Livelihood Science and Technology in Suzhou (SS2019061); Discipline Construction Project of the Second Affiliated Hospital of Soochow University (XKTJ-XK202004); Scientific Program of Suzhou Municipal Health and Health Committee (LCZX202004).

## Conflict of Interest

The authors declare that the research was conducted in the absence of any commercial or financial relationships that could be construed as a potential conflict of interest.

## Supplementary Material

Supplementary Figure 1 General flowchart and TME infiltrates of LUAD samples in our study. **(A)** Overview of the study design; **(B)** The specific 23 immune and stromal fractions represented by various colors in each LUAD sample from The Cancer Genome Atlas (TCGA) database.

Supplementary Figure 2 Determination and assessment of different TMEclusters. **(A, B)** Elbow method and gap statistic to determining the optimal number of clusters in the dataset; **(C)** Consensus matrix of the LUAD cohort for *K*=3, displaying the clustering stability using 1000 iterations of hierarchical clustering; **(D)** Kaplan-Meier curves showing the survival stratified by different TMEclusters.

Supplementary Figure 3 Characterization of DEGs among the three TMEclusters and the derived TME signature genes. **(A)** Venn diagram illustrating the number of differentially expressed genes (DEGs) among the three TMEclusters; **(B)** Consensus matrix of DEGs for *K*=2, displaying the clustering stability using 1000 iterations of hierarchical clustering; **(C)** Gene Ontology (GO) enrichment analysis of the TME relevant signature genes. The x axis indicates the number of genes within each GO term.

Supplementary Figure 4 Survival analyses of LUAD patients in different datasets stratified by TMEscore. **(A, B)** Kaplan-Meier curves exhibiting the survival of patient cohort from TCGA dataset with stage I-II disease stratified by TMEscore; **(C)** Kaplan-Meier curves for the two subgroups of patients from the Gene Expression Omnibus (GEO) dataset (GSE68465) stratified by TMEscore; **(D)** Kaplan-Meier curves for the survival of smokers in the GEO dataset stratified by TMEscore.

Supplementary Figure 5 Summary of the mutation information with statistical calculations. **(A–C)** Classification of mutation types according to different categories, in which missense mutation accounts for the most fraction of SNV, SNV showed more frequency than insertion or deletion, and C>A was the most common of SNV; **(D–E)** tumor mutation burden and variant classification in specific samples; **(F)** the top 10 mutated genes in LUADs.

Supplementary Figure 6 Mutation landscape of the top 10 mutated genes in TCGA LUAD cohort stratified by TME subtypes and its association with histologic subtypes.

Supplementary Figure 7 Survival curves of patient cohorts stratified by TME-related signature genes. **(A–E)** Kaplan-Meier curve showing OS of TCGA cohort stratified by **(A)** BTK, **(B)** LY86, **(C)** CCDC69, **(D)** CD33, and **(E)** CD52 expression, respectively; **(F)** Kaplan-Meier curve showing OS of domestic cohort stratified by BTK expression.

## References

[1] TCGA Research Network. Comprehensive molecular profiling of lung adenocarcinoma. Nature (2014) 511:543–50. 10.1038/nature13385 PMC4231481 25079552

[2] Warth A Muley T Meister M Stenzinger A Thomas M Schirmacher P. The Novel Histologic International Association for the Study of Lung Cancer/American Thoracic Society/European Respiratory Society Classification System of Lung Adenocarcinoma Is a Stage-Independent Predictor of Survival. J Clin Oncol (2012) 30:1438–46. 10.1200/JCO.2011.37.2185 22393100

[3] Buttner R Gosney JR Skov BG Adam J Motoi N Bloom KJ. Programmed Death-Ligand 1 Immunohistochemistry Testing: A Review of Analytical Assays and Clinical Implementation in Non-Small-Cell Lung Cancer. J Clin Oncol (2017) 35:3867–76. 10.1200/JCO.2017.74.7642 29053400

[4] Dong ZY Zhong WZ Zhang XC Su J Xie Z Liu SY. Potential Predictive Value of TP53 and KRAS Mutation Status for Response to PD-1 Blockade Immunotherapy in Lung Adenocarcinoma. Clin Cancer Res (2017) 23:3012–24. 10.1158/1078-0432.CCR-16-2554 28039262

[5] Reck M Rodriguez-Abreu D Robinson AG Hui R Csoszi T Fulop A. Pembrolizumab versus Chemotherapy for PD-L1-Positive Non-Small-Cell Lung Cancer. N Engl J Med (2016) 375:1823–33. 10.1056/NEJMoa1606774 27718847

[6] Naidoo J Page DB Li BT Connell LC Schindler K Lacouture ME. Toxicities of the anti-PD-1 and anti-PD-L1 immune checkpoint antibodies. Ann Oncol (2015) 26:2375–91. 10.1093/annonc/mdv383 PMC6267867 26371282

[7] Matikas A Zerdes I Lovrot J Richard F Sotiriou C Bergh J. Prognostic implications of PD-L1 expression in breast cancer: systematic review and meta-analysis of immunohistochemistry and pooled analysis of transcriptomic data. Clin Cancer Res (2019) 25:5717–26. 10.1158/1078-0432.CCR-19-1131 31227501

[8] Zeng D Li M Zhou R Zhang J Sun H Shi M. Tumor microenvironment characterization in gastric cancer identifies prognostic and immunotherapeutically relevant gene signatures. Cancer Immunol Res (2019) 7:737–50. 10.1158/2326-6066.CIR-18-0436 30842092

[9] Remark R Lupo A Alifano M Biton J Ouakrim H Stefani A. Immune contexture and histological response after neoadjuvant chemotherapy predict clinical outcome of lung cancer patients. Oncoimmunology (2016) 5:e1255394. 10.1080/2162402X.2016.1255394 28123901 PMC5213838

[10] Thommen DS Schreiner J Muller P Herzig P Roller A Belousov A. Progression of Lung Cancer Is Associated with Increased Dysfunction of T Cells Defined by Coexpression of Multiple Inhibitory Receptors. Cancer Immunol Res (2015) 3:1344–55. 10.1158/2326-6066.CIR-15-0097 26253731

[11] Zhang C Zhang J Xu FP Wang YG Xie Z Su J. Genomic Landscape and Immune Microenvironment Features of Preinvasive and Early-Invasive Lung Adenocarcinoma. J Thorac Oncol (2019) 14:1912–23. 10.1016/j.jtho.2019.07.031 PMC6986039 31446140

[12] Lavin Y Kobayashi S Leader A Amir ED Elefant N Bigenwald C. Innate Immune Landscape in Early Lung Adenocarcinoma by Paired Single-Cell Analyses. Cell (2017) 169:750–65.e17. 10.1016/j.cell.2017.04.014 28475900 PMC5737939

[13] Suda K Kim J Murakami I Rozeboom L Shimoji M Shimizu S. Innate Genetic Evolution of Lung Cancers and Spatial Heterogeneity: Analysis of Treatment-Naïve Lesions. J Thorac Oncol (2018) 13:1496–507. 10.1016/j.jtho.2018.05.039 PMC6153034 29933065

[14] Chen H Carrot-Zhang J Zhao Y Hu H Freeman SS Yu S. Genomic and immune profiling of pre-invasive lung adenocarcinoma. Nat Commun (2019) 10:5472. 10.1038/s41467-019-13460-3 31784532 PMC6884501

[15] Kinoshita T Kudo-Saito C Muramatsu R Fujita T Saito M Nagumo H. Determination of poor prognostic immune features of tumour microenvironment in non-smoking patients with lung adenocarcinoma. Eur J Cancer (2017) 86:15–27. 10.1016/j.ejca.2017.08.026 28950145

[16] Sakai T Aokage K Neri S Nakamura H Nomura S Tane K. Link between tumor-promoting fibrous microenvironment and an immunosuppressive microenvironment in stage I lung adenocarcinoma. Lung Cancer (2018) 126:64–71. 10.1016/j.lungcan.2018.10.021 30527194

[17] Donnem T Kilvaer TK Andersen S Richardsen E Paulsen EE Hald SM. Strategies for clinical implementation of TNM-Immunoscore in resected nonsmall-cell lung cancer. Ann Oncol (2016) 27:225–32. 10.1093/annonc/mdv560 26578726

[18] Corredor G Wang X Zhou Y Lu C Fu P Syrigos KN. Spatial architecture and arrangement of tumor-infiltrating lymphocytes for predicting likelihood of recurrence in early-stage non-small cell lung cancer. Clin Cancer Res (2019) 25:1526–34. 10.1158/1078-0432.CCR-18-2013 PMC6397708 30201760

[19] Krysan K Tran LM Grimes BS Fishbein GA Seki A Gardner BK. The Immune Contexture Associates with the Genomic Landscape in Lung Adenomatous Premalignancy. Cancer Res (2019) 79:5022–33. 10.1158/0008-5472.CAN-19-0153 PMC6774823 31142513

[20] Newman AM Liu CL Green MR Gentles AJ Feng W Xu Y. Robust enumeration of cell subsets from tissue expression profiles. Nat Methods (2015) 12:453–7. 10.1038/nmeth.3337 PMC4739640 25822800

[21] Becht E Giraldo NA Lacroix L Buttard B Elarouci N Petitprez F. Estimating the population abundance of tissue-infiltrating immune and stromal cell populations using gene expression. Genome Biol (2016) 17:218. 10.1186/s13059-016-1070-5 27765066 PMC5073889

[22] Gautier L Cope L Bolstad B Irizarry R. affy–analysis of Affymetrix GeneChip data at the probe level. Bioinformatics (2004) 20:307–15. 10.1093/bioinformatics/btg405 14960456

[23] Jabi M Pedersoli M Mitiche A Ben AI. Deep clustering: On the link between discriminative models and K-means. IEEE Trans Pattern Anal Mach Intell (2019). 10.1109/TPAMI.2019.2962683 31899413

[24] Monti S Tamayo P Mesirov J Golub T. Consensus Clustering: A Resampling-Based Method for Class Discovery and Visualization of Gene Expression Microarray Data. Mach Learn (2003) 52:91–118. 10.1023/A:1023949509487

[25] Ritchie ME Phipson B Wu D Hu Y Law CW Shi W. limma powers differential expression analyses for RNA-sequencing and microarray studies. Nucleic Acids Res (2015) 43:e47. 10.1093/nar/gkv007 25605792 PMC4402510

[26] Yu G Wang L-G Han Y He Q-Y. clusterProfiler: an R package for comparing biological themes among gene clusters. OMICS (2012) 16:284–7. 10.1089/omi.2011.0118 PMC3339379 22455463

[27] Hothorn T. maxstat: Maximally Selected Rank Statistics. R package version 0.7-22. (2015). 10.1158/2326-6066.cir-18-0436

[28] Mayakonda A Lin DC Assenov Y Plass C Koeffler HP. Maftools: efficient and comprehensive analysis of somatic variants in cancer. Genome Res (2018) 28:1747–56. 10.1101/gr.239244.118 PMC6211645 30341162

[29] Gehring JS Fischer B Lawrence M Huber W. SomaticSignatures: inferring mutational signatures from single-nucleotide variants. Bioinformatics (2015) 31:3673–5. 10.1093/bioinformatics/btv408 PMC4817139 26163694

[30] Alexandrov LB Nik-Zainal S Wedge DC Aparicio SA Behjati S Biankin AV. Signatures of mutational processes in human cancer. Nature (2013) 500:415–21. 10.1038/nature12477 PMC3776390 23945592

[31] Alexandrov LB Nik-Zainal S Wedge DC Campbell PJ Stratton MR. Deciphering signatures of mutational processes operative in human cancer. Cell Rep (2013) 3:246–59. 10.1016/j.celrep.2012.12.008 PMC3588146 23318258

[32] van Dessel L van Riet J Smits M Zhu Y Hamberg P van der Heijden M. The genomic landscape of metastatic castration-resistant prostate cancers reveals multiple distinct genotypes with potential clinical impact. Nat Commun (2019) 10:5251. 10.1038/s41467-019-13084-7 31748536 PMC6868175

[33] Jiang P Gu S Pan D Fu J Sahu A Hu X. Signatures of T cell dysfunction and exclusion predict cancer immunotherapy response. Nat Med (2018) 24:1550–8. 10.1038/s41591-018-0136-1 PMC6487502 30127393

[34] Wang Q Li M Yang M Yang Y Song F Zhang W. Analysis of immune-related signatures of lung adenocarcinoma identified two distinct subtypes: implications for immune checkpoint blockade therapy. Aging (2020) 12:3312–39. 10.18632/aging.102814 PMC7066911 32091408

[35] Wang S He Z Wang X Li H Liu XS. Antigen presentation and tumor immunogenicity in cancer immunotherapy response prediction. eLife (2019) 8:e49020. 10.7554/eLife.49020 31767055 PMC6879305

[36] Keenan TE Burke KP Van Allen EM. Genomic correlates of response to immune checkpoint blockade. Nat Med (2019) 25:389–402. 10.1038/s41591-019-0382-x 30842677 PMC6599710

[37] Kaderbhaï C Tharin Z Ghiringhelli F. The Role of Molecular Profiling to Predict the Response to Immune Checkpoint Inhibitors in Lung Cancer. Cancers (2019) 11:201. 10.3390/cancers11020201 PMC6406957 30744168

[38] Geeleher P Cox N Huang RS. pRRophetic: an R package for prediction of clinical chemotherapeutic response from tumor gene expression levels. PloS One (2014) 9:e107468. 10.1371/journal.pone.0107468 25229481 PMC4167990

[39] Qiu X Chen D Liu Y Duan S Zhang F Zhang Y. Relationship between stromal cells and tumor spread through air spaces in lung adenocarcinoma. Thorac Cancer (2019) 10:256–67. 10.1111/1759-7714.12945 PMC6360243 30605235

[40] Benjamini Y Hochberg Y. Controlling the false discovery rate: a practical and powerful approach to multiple testing. J R Stat Soc: Ser B (Methodological) (1995) 57:289–300. 10.1111/j.2517-6161.1995.tb02031.x

[41] Robin X Turck N Hainard A Tiberti N Lisacek F Sanchez J-C. pROC: an open-source package for R and S+ to analyze and compare ROC curves. BMC Bioinf (2011) 12:77. 10.1186/1471-2105-12-77 PMC3068975 21414208

[42] Hause RJ Pritchard CC Shendure J Salipante SJ. Classification and characterization of microsatellite instability across 18 cancer types. Nat Med (2016) 22:1342–50. 10.1038/nm.4191 27694933

[43] Luchini C Bibeau F Ligtenberg MJL Singh N Nottegar A Bosse T. ESMO recommendations on microsatellite instability testing for immunotherapy in cancer, and its relationship with PD-1/PD-L1 expression and tumour mutational burden: a systematic review-based approach. Ann Oncol (2019) 30:1232–43. 10.1093/annonc/mdz116 31056702

[44] Fu J Li K Zhang W Wan C Zhang J Jiang P. Large-scale public data reuse to model immunotherapy response and resistance. Genome Med (2020) 12:21. 10.1186/s13073-020-0721-z 32102694 PMC7045518

[45] Yue C Ma H Zhou Y. Identification of prognostic gene signature associated with microenvironment of lung adenocarcinoma. PeerJ (2019) 7:e8128. 10.7717/peerj.8128 31803536 PMC6886493

[46] Bi KW Wei XG Qin XX Li B. BTK Has Potential to Be a Prognostic Factor for Lung Adenocarcinoma and an Indicator for Tumor Microenvironment Remodeling: A Study Based on TCGA Data Mining. Front Oncol (2020) 10:424:424. 10.3389/fonc.2020.00424 32351880 PMC7175916

[47] Tan Q Huang Y Deng K Lu M Wang L Rong Z. Identification immunophenotyping of lung adenocarcinomas based on the tumor microenvironment. J Cell Biochem (2020) 121:4569–79. 10.1002/jcb.29675 32030808

[48] Huang J Li J Zheng S Lu Z Che Y Mao S. Tumor microenvironment characterization identifies two lung adenocarcinoma subtypes with specific immune and metabolic state. Cancer Sci (2020) 111(6):1876–86. 10.1111/cas.14390 PMC7293093 32187778

[49] Song Q Shang J Yang Z Zhang L Zhang C Chen J. Identification of an immune signature predicting prognosis risk of patients in lung adenocarcinoma. J Transl Med (2019) 17:70. 10.1186/s12967-019-1824-4 30832680 PMC6399972

[50] Zhang M Zhu K Pu H Wang Z Zhao H Zhang J. An Immune-Related Signature Predicts Survival in Patients With Lung Adenocarcinoma. Front Oncol (2019) 9:1314. 10.1186/s12967-019-1824-4 31921619 PMC6914845

[51] Hung JJ Wu YC Chou TY Jeng WJ Yeh YC Hsu WH. Adjuvant Chemotherapy Improves the Probability of Freedom From Recurrence in Patients With Resected Stage IB Lung Adenocarcinoma. Ann thoracic Surg (2016) 101:1346–53. 10.1016/j.athoracsur.2015.10.075 26794883

[52] Qian F Yang W Wang R Xu J Wang S Zhang Y. Prognostic significance and adjuvant chemotherapy survival benefits of a solid or micropapillary pattern in patients with resected stage IB lung adenocarcinoma. J Thorac Cardiovasc Surg (2018) 155:1227–35.e2. 10.1016/j.jtcvs.2017.09.143 29223834

[53] Wang J Wu N Lv C Yan S Yang Y. Should patients with stage IB non-small cell lung cancer receive adjuvant chemotherapy? A comparison of survival between the 8th and 7th editions of the AJCC TNM staging system for stage IB patients. J Cancer Res Clin Oncol (2019) 145:463–9. 10.1007/s00432-018-2801-7 PMC11810431 30474757

