# Supplementary figures and images for: Characterization of Tumor Microenvironment in Lung Adenocarcinoma Identifies Immune Signatures to Predict Clinical Outcomes and Therapeutic Responses
Source: Front Oncol. 2021 Mar 5;11:581030. doi: 10.3389/fonc.2021.581030 (PMC7973234; doi:10.3389/fonc.2021.581030)

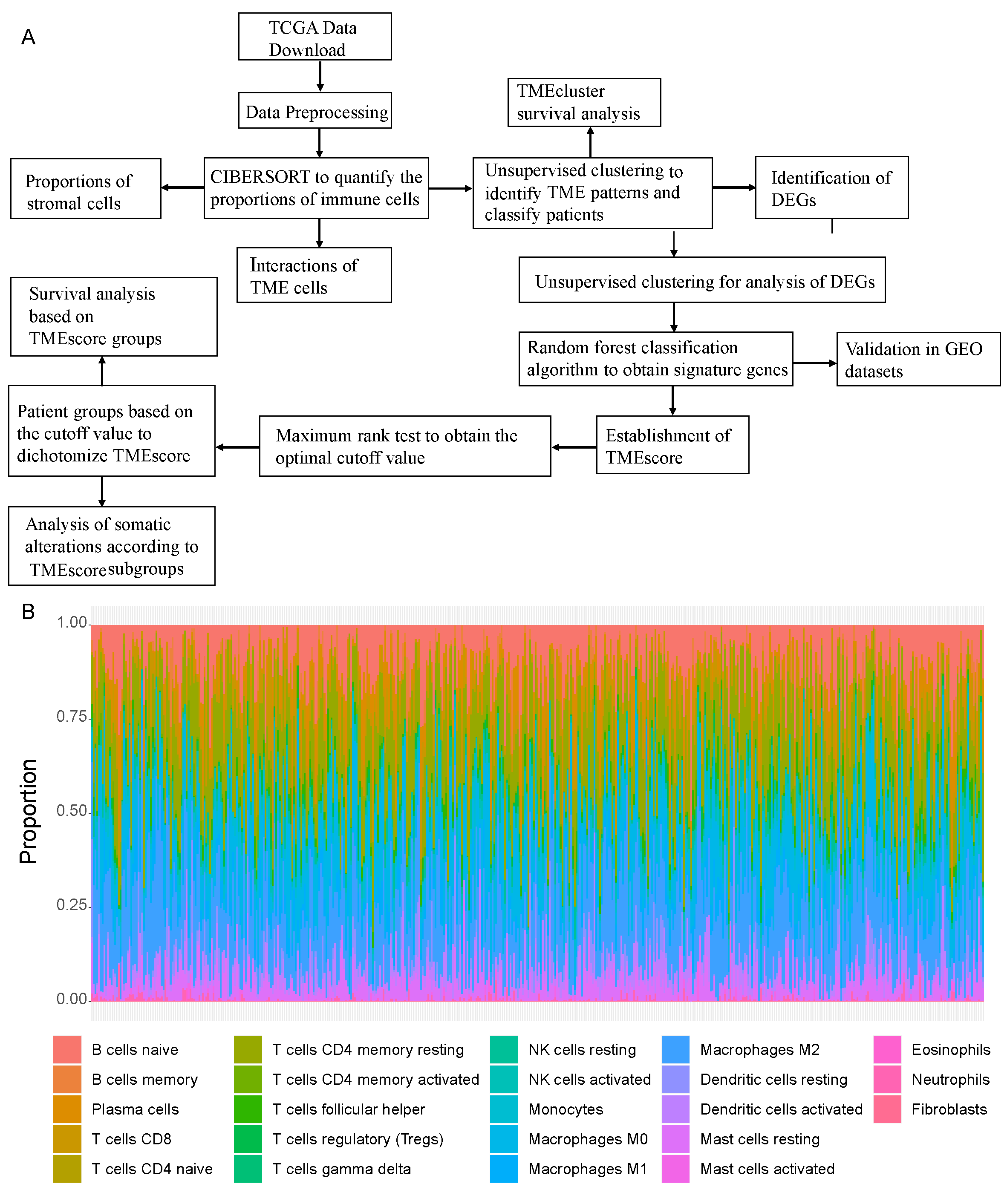

Supplement: Supplementary Figure 1 — General flowchart and TME infiltrates of LUAD samples in our study. (A) Overview of the study design; (B) The specific 23 immune and stromal fractions represented by various colors in each LUAD sample from The Cancer Genome Atlas (TCGA) database. [file Image_1.tif]

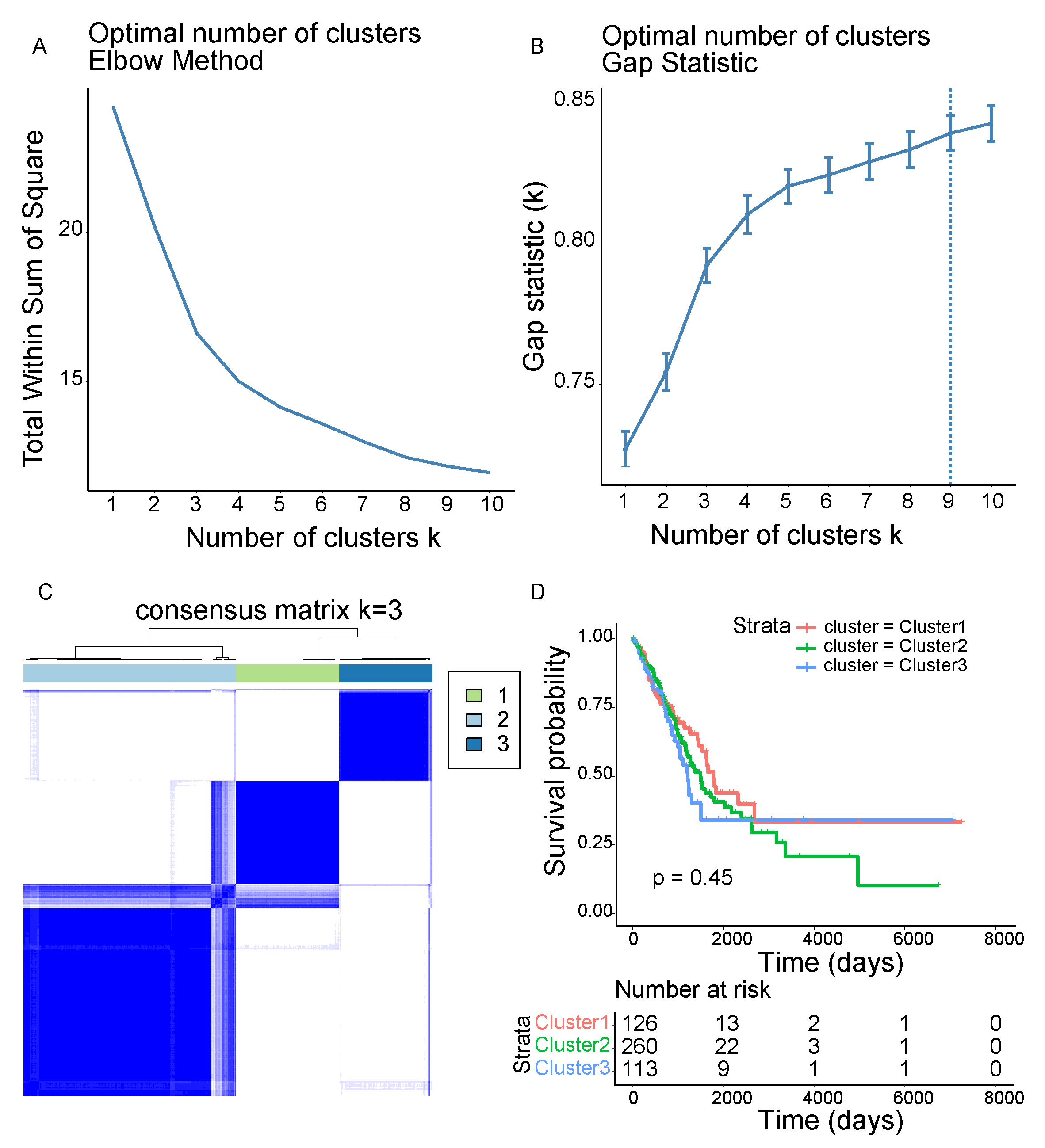

Supplement: Supplementary Figure 2 — Determination and assessment of different TMEclusters. (A, B) Elbow method and gap statistic to determining the optimal number of clusters in the dataset; (C) Consensus matrix of the LUAD cohort for K=3, displaying the clustering stability using 1000 iterations of hierarchical clustering; (D) Kaplan-Meier curves showing the survival stratified by different TMEclusters. [file Image_2.tif]

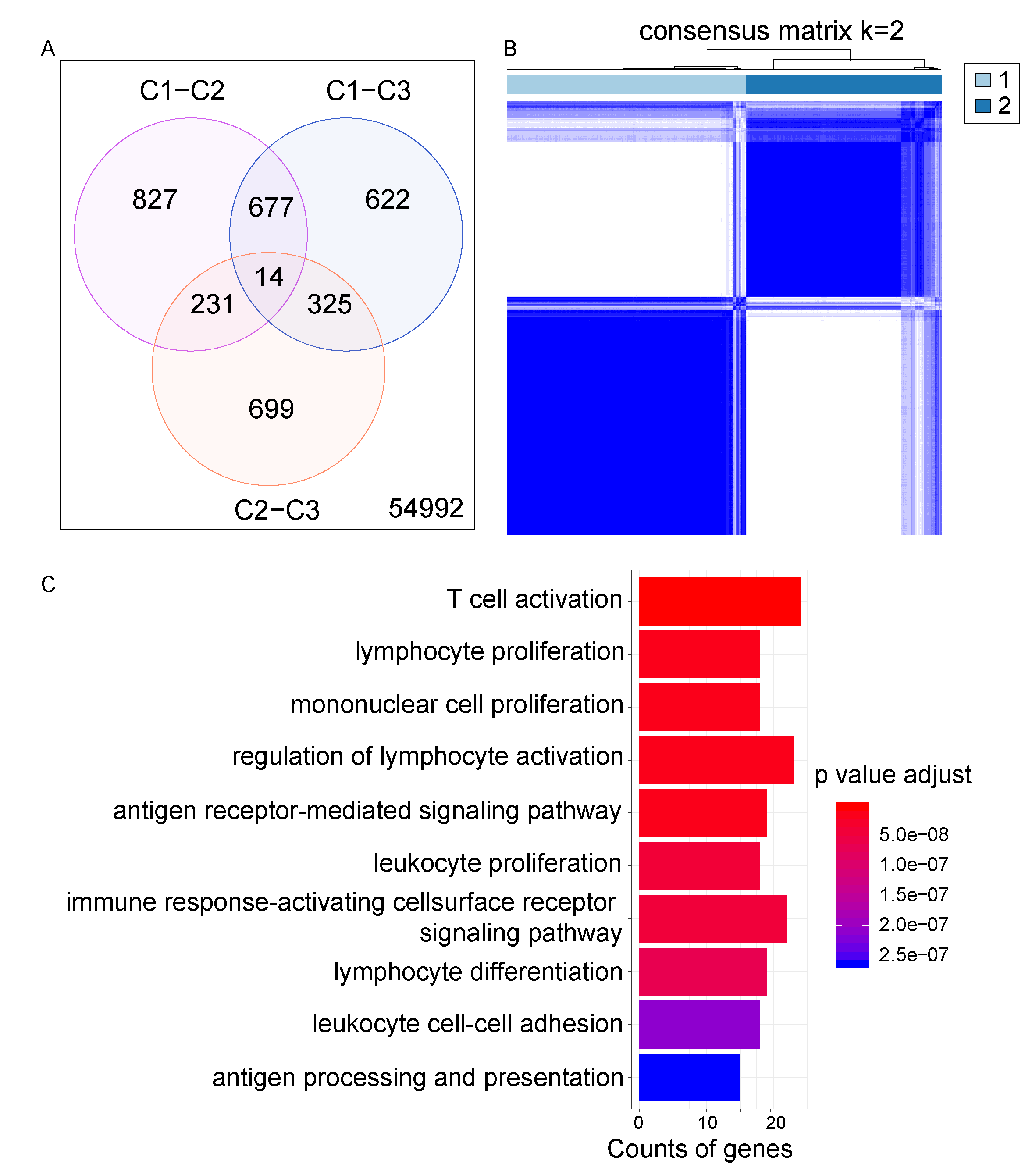

Supplement: Supplementary Figure 3 — Characterization of DEGs among the three TMEclusters and the derived TME signature genes. (A) Venn diagram illustrating the number of differentially expressed genes (DEGs) among the three TMEclusters; (B) Consensus matrix of DEGs for K=2, displaying the clustering stability using 1000 iterations of hierarchical clustering; (C) Gene Ontology (GO) enrichment analysis of the TME relevant signature genes. The x axis indicates the number of genes within each GO term. [file Image_3.tif]

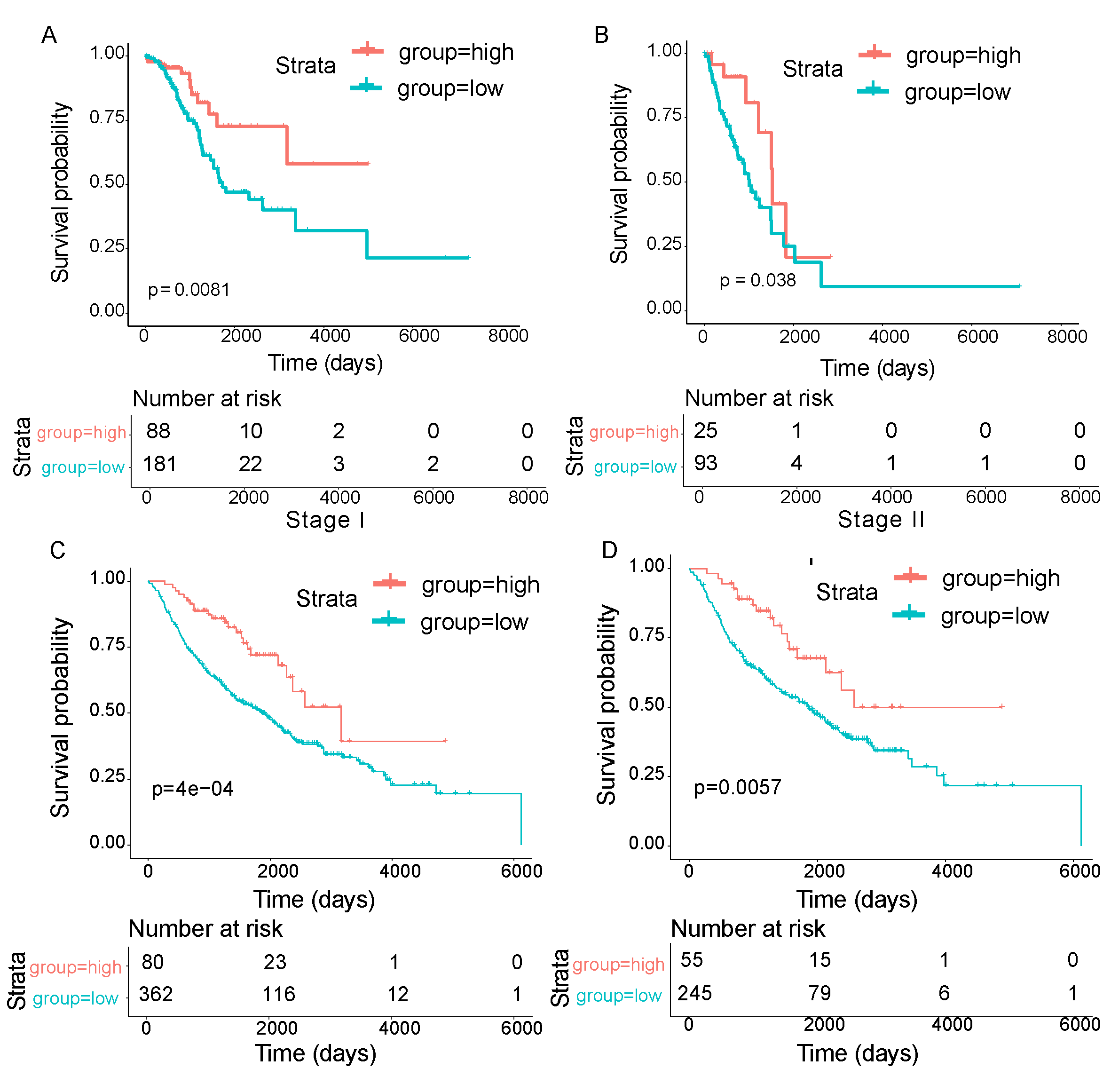

Supplement: Supplementary Figure 4 — Survival analyses of LUAD patients in different datasets stratified by TMEscore. (A, B) Kaplan-Meier curves exhibiting the survival of patient cohort from TCGA dataset with stage I-II disease stratified by TMEscore; (C) Kaplan-Meier curves for the two subgroups of patients from the Gene Expression Omnibus (GEO) dataset (GSE68465) stratified by TMEscore; (D) Kaplan-Meier curves for the survival of smokers in the GEO dataset stratified by TMEscore. [file Image_4.tif]

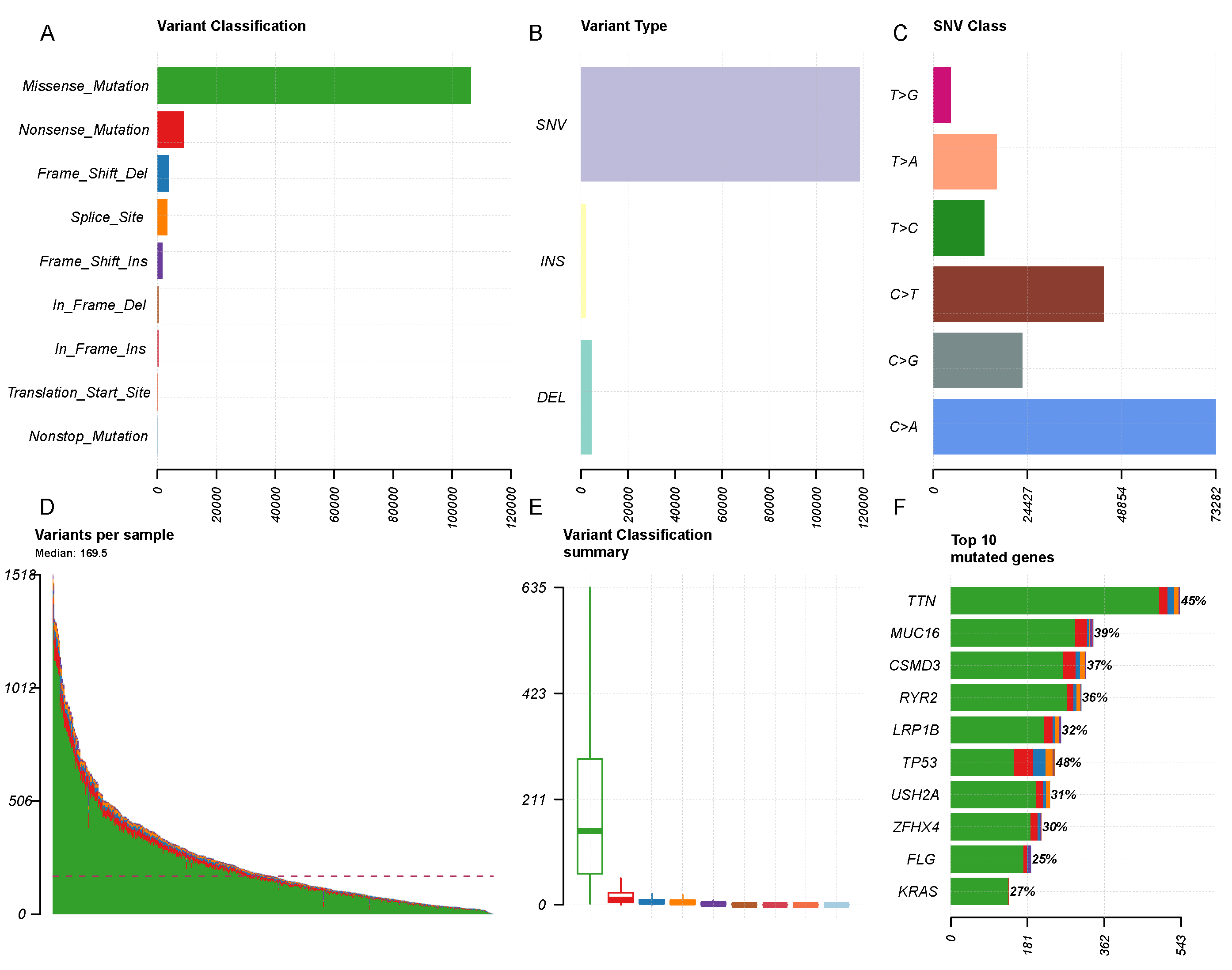

Supplement: Supplementary Figure 5 — Summary of the mutation information with statistical calculations. (A–C) Classification of mutation types according to different categories, in which missense mutation accounts for the most fraction of SNV, SNV showed more frequency than insertion or deletion, and C>A was the most common of SNV; (D–E) tumor mutation burden and variant classification in specific samples; (F) the top 10 mutated genes in LUADs. [file Image_5.tif]

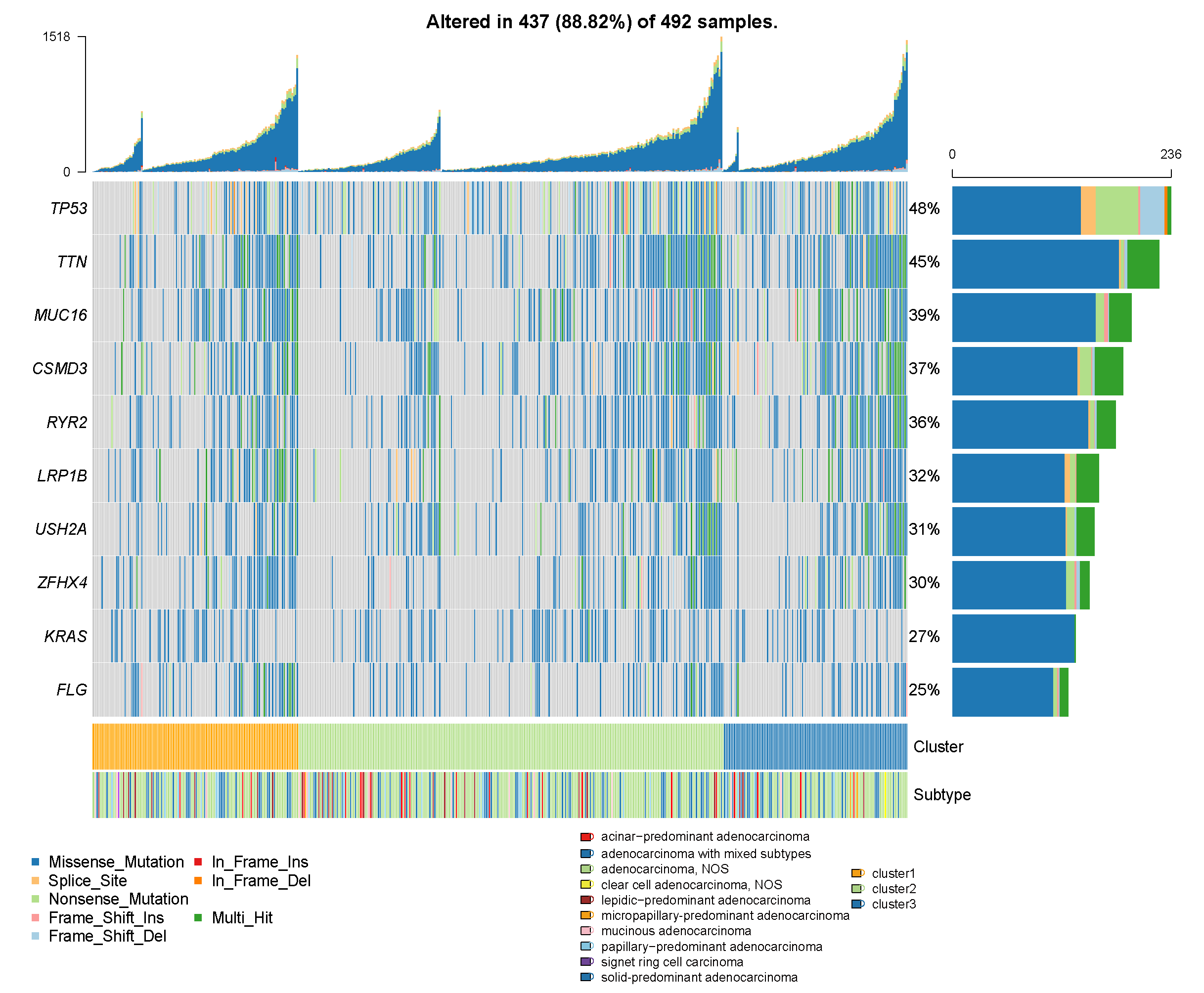

Supplement: Supplementary Figure 6 — Mutation landscape of the top 10 mutated genes in TCGA LUAD cohort stratified by TME subtypes and its association with histologic subtypes. [file Image_6.tif]

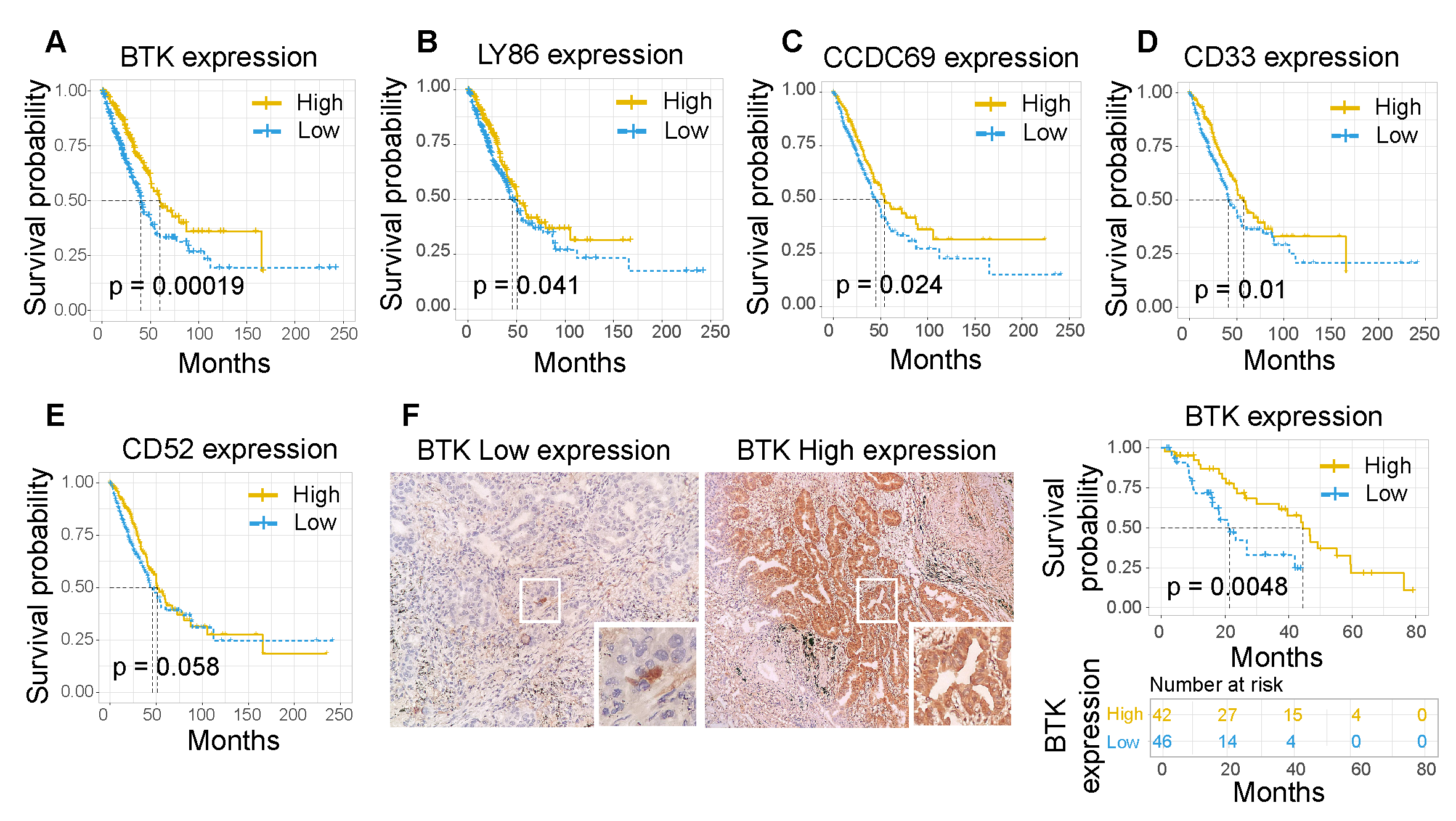

Supplement: Supplementary Figure 7 — Survival curves of patient cohorts stratified by TME-related signature genes. (A–E) Kaplan-Meier curve showing OS of TCGA cohort stratified by (A) BTK, (B) LY86, (C) CCDC69, (D) CD33, and (E) CD52 expression, respectively; (F) Kaplan-Meier curve showing OS of domestic cohort stratified by BTK expression. [file Image_7.tif]
