# Supplementary material for: Characterization of Tumor Microenvironment in Lung Adenocarcinoma Identifies Immune Signatures to Predict Clinical Outcomes and Therapeutic Responses
Source: Front Oncol. 2021 Mar 5;11:581030. doi: 10.3389/fonc.2021.581030 (PMC7973234; doi:10.3389/fonc.2021.581030)
Supplement: Supplementary file 8 [file DataSheet_1.docx]

|  | | Supplemental Table 1. Baseline information of the lung adenocarcinoma datasets | | | | | | |
| --- | --- | --- | --- | --- | --- | --- | --- | --- |
| Series accession numbers | Platform used | | Number of patients | AJCC_Stage | Gender | Mean age  [min, max] | Region | Survival outcome |
| TCGA-LUAD | Illumina RNAseq | | 499 | I: 269 II: 118 III: 80 IV: 25 | Male: 230 Female: 269 | 65.2[33, 88] | NA | Overall Survival |
| GSE68465 | Affymetrix Human Genome U133A Array | | 442 | NA | Male: 223 Female: 219 | 64.4[33, 87] | USA | Overall Survival |

| Supplemental Table 2. Baseline information of the lung adenocarcinoma cohort from the Second Affiliated Hospital of Soochow University | |
| --- | --- |
| Clinicopathological Characteristics | Number of patients (n = 88) |
| Gender(Male/Female) | 40/48 |
| Smoking history(Yes/No) | 25/53 |
| Surgical procedures(Lobectomy/Sublobar resection) | 72/16 |
| Surgical approaches(Thoracotomy/Video-assisted thoracoscopic surgery) | 8/80 |
| Pathological stage(I/II/IIIA) | 32/21/35 |
| Pathological Grades(High/Medium/Low) | 29/42/17 |
| Adjuvant treatment(Yes/No) | 58/33 |

| Supplemental Table 3. Effect of different cell types on overall survival | | | | | |
| --- | --- | --- | --- | --- | --- |
| Cell types | *p* value | Hazard ratio | Lower 95% Confidence Interval | Upper 95% Confidence Interval | Cluster |
| B cells naive | 0.27443 | 1.177029 | 0.87825 | 1.577453 | A |
| Plasma cells | 0.020777 | 1.414237 | 1.052641 | 1.900045 | A |
| B cells memory | 0.256495 | 1.253961 | 0.847231 | 1.855951 | B |
| T cells CD8 | 0.976231 | 0.995604 | 0.743215 | 1.333701 | B |
| T cells CD4 naive | 0.676991 | 0.809515 | 0.300136 | 2.183394 | B |
| T cells CD4 memory activated | 0.613567 | 0.926773 | 0.689871 | 1.245027 | B |
| T cells follicular helper | 0.382731 | 0.877321 | 0.653844 | 1.17718 | B |
| T cells regulatory (Tregs) | 0.115744 | 0.790234 | 0.588859 | 1.060473 | B |
| T cells gamma delta | 0.647348 | 1.304986 | 0.414362 | 4.109912 | B |
| NK cells resting | 0.091452 | 1.287632 | 0.959454 | 1.728063 | B |
| NK cells activated | 0.698849 | 0.943906 | 0.704555 | 1.264568 | B |
| Monocytes | 0.014726 | 1.441572 | 1.072806 | 1.937098 | B |
| Macrophages M1 | 0.197662 | 0.825161 | 0.615656 | 1.105959 | B |
| Dendritic cells resting | 0.126243 | 1.261036 | 0.936161 | 1.698652 | B |
| Dendritic cells activated | 0.927364 | 0.98653 | 0.736413 | 1.321598 | B |
| Mast cells resting | 0.188989 | 1.217271 | 0.907422 | 1.63292 | B |
| Mast cells activated | 0.007552 | 0.557181 | 0.360647 | 0.860817 | B |
| Eosinophils | 0.776157 | 1.11609 | 0.523847 | 2.3779 | B |
| Neutrophils | 0.249076 | 0.84097 | 0.626333 | 1.129161 | B |
| Fibroblasts | 0.262573 | 0.84586 | 0.630865 | 1.134125 | B |
| T cells CD4 memory resting | 0.854789 | 1.027777 | 0.766907 | 1.377386 | C |
| Macrophages M0 | 0.07369 | 0.765333 | 0.570374 | 1.02693 | C |
| Macrophages M2 | 0.746619 | 0.952837 | 0.710798 | 1.277295 | C |
